# Effect of Mixed Recycled Aggregate on the Mechanical Strength and Microstructure of Concrete under Different Water Cement Ratios

**DOI:** 10.3390/ma14102631

**Published:** 2021-05-18

**Authors:** Tao Meng, Huadong Wei, Xiufen Yang, Bo Zhang, Yuncai Zhang, Cungui Zhang

**Affiliations:** 1 College of Civil Engineering and Architecture, Zhejiang University, Hangzhou 310058, China; 21812061@zju.edu.cn (H.W.); 22012059@zju.edu.cn (X.Y.); 2 Ocean Research Center of Zhoushan, Zhejiang University, Zhoushan 316021, China; zb911@zju.edu.cn; 3 Zhoushan Jinke Resources Recycling Co., Ltd., Zhoushan 316000, China; 21912044@zju.edu.cn; 4 Shanxi Sixth Construction Group Co., Ltd., Taiyuan 030024, China; 21712037@zju.edu.cn

**Keywords:** mixed recycled aggregate, water–cement ratio, mechanical properties, hydration process, microstructure

## Abstract

Mixed recycled aggregate (MRA) is a kind of recycled aggregate containing discarded bricks and other impurities that is inferior to ordinary recycled concrete aggregate. To study the effect of MRA in concrete, specimens with 100% MRA under different water–cement ratios (W/C) of 0.50, 0.42, 0.36 and 0.30 were prepared, and the mechanical properties and microstructure were tested. Results show that compared with ordinary concrete, the compressive strength of mixed recycled aggregate concrete (MRAC) with the same W/C was reduced by more than 50% at 28 days, but the axial compression ratio was relatively high, reaching over 0.87. Affected by the high water absorption of MRA, the hydration rate of cement slowed, which was beneficial to the long-term development of the properties of MRAC. An appropriate increase in cement content could strengthen MRA and densify the pore structure of MRAC. The research results of this article prove that MRA has high utilization value and could be used to prepare MRAC with application potential using optimal gradation, which is of positive significance for promoting the consumption of construction waste.

## 1. Introduction

As a widely used building material, concrete consumes many natural resources, which has a negative impact on the environment. At the same time, the acceleration of urbanization and the demolition of old buildings generates significant quantities of construction waste. According to incomplete statistics, China’s annual construction waste is approximately 1.5 billion tons, resulting in a large amount of solid waste, including broken bricks, gravel, scrap iron, and ceramics [1]. Using a common landfill method to dispose of these solid wastes will not only occupy land space but pollute the soil, water, and air.

Regarding the issues above, some scholars considered crushing the industrial by-products and construction solid waste from which impurities have been removed into recycled aggregate (RA), then replacing natural aggregate partially or completely to prepare recycled aggregate concrete (RAC), holding that this can relieve resource pressure and effectively eliminate construction waste [2,3,4,5]. In general, the RA produced by the crushing process is unstable with cracks inside, and the density and crushing index will decrease to varying degrees, which can then be easily invaded by corrosive media [6,7]. The content of old mortar attached to RA normally exceeds 30% [8]. Therefore, compared with natural aggregate, the water absorption of RA is more significant, and the porosity, shape, specific surface area, etc. change [9,10,11]. Due to performance degradation, the properties will inevitably decrease when RA is used to prepare concrete [12]. The workability of the concrete mixture deteriorates due to the high water absorption of the RA, and the density of the mixture will also be reduced by 5–15% compared with natural aggregate concrete [8,13,14]. The total porosity of concrete greatly varies with the dry and saturated conditions of the RA [15]. The linear thermal expansion coefficient of concrete will increase due to the addition of recycled coarse aggregate [16]. Kwan et al. found that RAC would expand during water curing, and the higher the RA content, the higher the expansion would be [17]. In terms of mechanical properties, the use of RA will adversely affect the strength of concrete. The compressive strength could be reduced by 30% at a 100% replacement rate, and the average drop in split tensile strength could be up to 10% [18,19,20]. To increase the utilization of RAC, some approaches have been proposed. Shi et al. believed that pretreatment of RA by carbonization is environmentally friendly and effective [21]. The use of biotreatment or the incorporation of steel fiber and nanosilica can improve both the mechanical properties and microstructure of RAC [22,23,24,25].

At present, research on RAC has made much progress and could be applied to real project [26,27]. However, brick-concrete houses are common in old buildings, especially in China, where clay bricks occupy a large proportion of the wall materials [28]. It is estimated that China produces about 400 million tons of brick waste every year [29]. In addition, it would take considerable manpower and material resources to sort out all the broken bricks in the dismantled garbage, which increases the production cost of RA. Since clay brick is a kind of low-strength porous material, mixing it into RA and using it as a recycled raw material will further reduce the density and strength and increase the crushing index [12]. Discarded clay brick also leads to a high water absorption coefficient and affects the water–cement ratio (W/C) of concrete [30,31,32]. To avoid the absorption of RA to mixing water, Pacheco-Torgal and Anderson et al. soaked RA for 24 h then transformed it into a saturated dry surface state [33,34]. Gomes and De Brito adopted the prewetting method, which can also reduce the adverse effects of the high water absorption of RA [35,36,37]. 

Due to disadvantages such as complex composition, low density, high crushing index, and large water absorption, it is generally recommended that the content of recycled bricks in the aggregate should not exceed 20%, and the replacement ratio of mixed recycled aggregate (MRA) should not exceed 50% [38,39,40]. However, these low-replacement recycled products can only consume a limited amount of MRA and cannot meet the huge disposal needs of brick-concrete construction solid waste. Based on this, many scholars have carried out research on the utilization of MRA with high replacement rates. Aliabdo et al. found that mixing a high content of recycled bricks would reduce the elastic modulus and split tensile strength of concrete and increase the concrete porosity [41]. Nepomuceno et al. found that as the replacement rate of MRA increases the mechanical properties of concrete continuously decrease [31]. When the replacement rate reached 75%, the compressive strength decreased by 11.1%, the bending strength decreased by 5.8%, and the splitting tensile strength decreased by 22.2%. It is recommended that the replacement rate of MRA when used in building structures should not exceed 30%.

Through previous studies, it can be found that the main factor limiting the utilization of MRA is the unclear effect of MRA on the properties of concrete, especially the strength development of concrete under different W/C ratios. Based on this, this paper designed and prepared four mixed recycled aggregate concretes (MRACs) under different W/C ratios of 0.30, 0.36, 0.42, and 0.50. The effects of W/C and age on the strength of MRAC were studied through macroscopic mechanical property tests. Mercury intrusion porosimetry (MIP), X-ray diffraction (XRD), and scanning electron microscopy (SEM) were also used to verify the general influence of different W/C ratios on the mechanical properties of MRAC.

## 2. Materials and Experimental Methods

### 2.1. Materials

**Figure 1:**
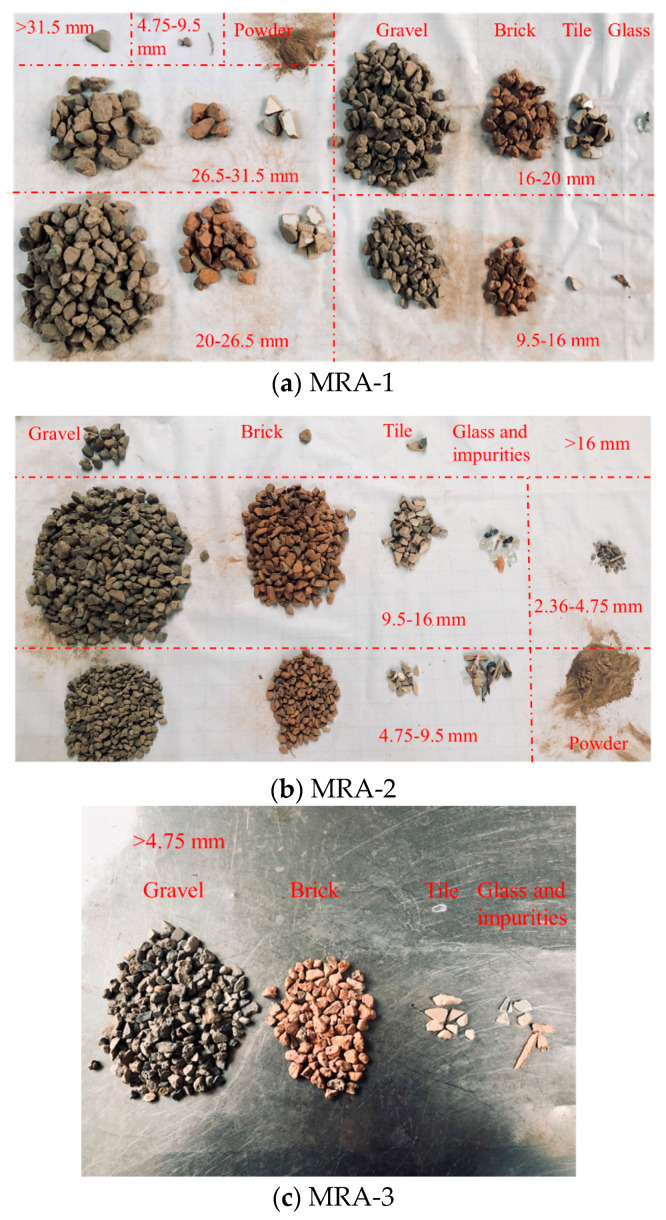
The basic compositions of three MRAs.

**Figure 2:**
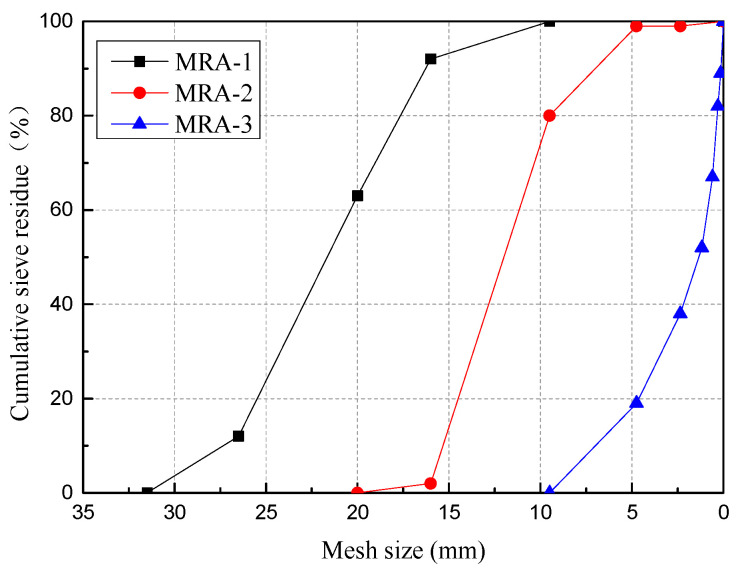
Gradation curve of MRAs.

The MRA used in this study was provided by Zhoushan Jinke Resources Recycling Co., Ltd. (Zhoushan, China) The aggregate is divided into three fractions. The first fraction is MRA-1 with a particle size of approximately 18–32 mm; the second fraction is MRA-2 with a particle size of 10–18 mm; the last fraction is MRA-3 with a particle size of 0–10 mm and a fineness modulus of 2.9. The basic compositions of the three MRAs are shown in Figure 1. According to the Chinese standards of Recycled Coarse Aggregate for Concrete (GB/T 25177-2010) and Recycled Fine Aggregate for Concrete and Mortar (GB/T 25176-2010), the main technical indicators of the three types of MRAs are shown in Table 1, and the particle grading curve is shown in Figure 2.

Standard cement (P.I 42.5) was used as the cementing material. According to the Chinese standard of Standard Cement Technical Conditions for Properties Inspection of Concrete Admixtures (GB 8076-2008), the basic properties of standard cement are shown in Table 2. The water reducing agent is a polycarboxylic acid type with a water reducing rate of 10%; the water is tap water from the laboratory.

### 2.2. Mix Design Method

**Figure 3:**
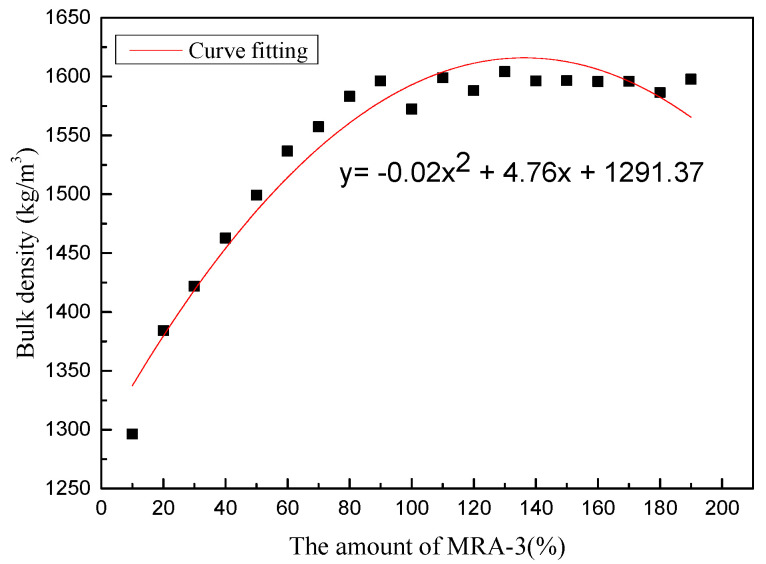
The Bulk Density of MRA.

There are three grades of MRAs used in this article. First, the mixing ratio of MRA-1 and MRA-2 was determined by the closest packing method. The results showed that the bulk density of MRA-1 and MRA-2 reached a maximum value when the mixing amount of MRA-2 was 15%. Then, MRA-3 was added in equal proportions to the mixed aggregate, and the bulk density curve is shown in Figure 3. The results showed that the bulk density of MRA fluctuates within a certain range when the amount of MRA-3 blended exceeds 90%. To control the rate of fine aggregate, the fitted curve from the data points was used to determine the amount of MRA-3, and the maximum mixing amount was at the point of 140%. The final mixing ratio of the three grades of MRA is 36.2:5.5:58.3. According to the mixing ratio, MRAC under different W/C ratios of 0.50, 0.42, 0.36, and 0.30 were prepared, in which the amount of water reducing agent was 1.8% of the mass of the cement. According to previous research [7], the additional water consumption was the 2 h water absorption of MRA and not included for the calculation of W/C. The slump is controlled within 150 ± 30 mm. The mix ratio is presented in Table 3.

### 2.3. Mechanical Properties Test

The test samples were cured in a standard curing room with a temperature of 20 ± 2 °C and a relative humidity of more than 95% after forming. The compressive strength and splitting tensile strength of concrete samples with 100% MRA corresponding to ages of 3 d, 7 d and 28 d were tested according to the Standard for Test Methods of Concrete Physical and Mechanical Properties (GB/T 50081-2019). The axial compressive strength and elastic modulus were tested at the age of 28 days according to the same standard of GB/T 50081-2019.

To compare with ordinary concrete, we calculated the compressive strength of ordinary concrete under the same W/C according to the Bolomy formula used in the Specification for Mix Proportion Design of Ordinary Concrete (JGJ 55-2011):

(1) $$f_{cu}=a_{a}f_{b}\left(\frac{C}{W}-a_{b}\right)$$

where $f_{cu}$ = cubic compressive strength of concrete (MPa); $\frac{C}{W}$ = cement-water ratio; $f_{b}$ = cement compressive strength at 28 days (MPa); $a_{a}$, $a_{b}$= empirical coefficient related to aggregate, and the recommended values are ($a_{a}=0.53$, $a_{b}=0.20$).

### 2.4. Mercury Intrusion Porosimetry

The Mercury Intrusion Porosimetry (MIP) method was used to determine the porosity and pore size distribution of MRAC. The test instrument was the AutoPore IV 9500 Mercury Porosimeter (Micromeritics, Dr Norcross, GA, USA) by the National Key Laboratory of Chemical Engineering, Zhejiang University. The samples near the center area of MRAC were broken into small fragments after the compressive strength test and dried before the MIP test.

### 2.5. X-ray Diffraction Analysis

X-ray Diffraction (XRD) Analysis technology was used to determine the mineral composition of MRAC under different W/C ratios at 28 d. The test instrument was a Bruker D8 ADVACNCE X-ray diffractometer (Bruker, Bremen, Germany) by the Analysis Center of Zhejiang University. The MRAC samples were ground into powder that could pass through a 0.08 mm square hole sieve before XRD analysis.

### 2.6. Scanning Electron Microscope Test

Scanning Electron Microscope (SEM) Test was used to analyze the influence of W/C on the pore structure and cement hydration products. The test instrument was a Gemini SEM300 thermal field emission scanning electron microscope (ZEISS, Jena, Germany) by the Analysis Center of Zhejiang University. The hydration of small fragment samples for SEM was stopped by absolute ethanol at a specific curing time. Then, those samples were sprayed with gold for 60 s to be conductive before SEM test.

## 3. Results and Discussion

### 3.1. Results of the Compressive Strength Test

**Figure 4:**
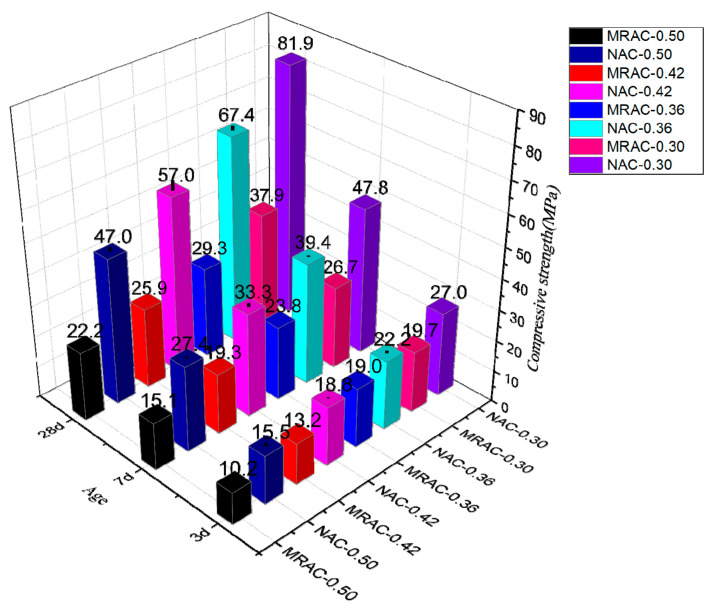
Comparison of Compressive Strength of MRAC with Reference Values. (Note: NAC-X represents the reference group under the same W/C as the MRAC).

Figure 4 showed that the compressive strength of each group of MRACs continuously improved with increasing age. Among these, the strength of group MRAC-0.50 has the most obvious increase. The 7-d compressive strength is 48.0% higher than that at 3 d, and the 28-d compressive strength is 47.0% higher than that at 7 d. The compressive strength increases of group MRAC-0.42 were second, the 7-d compressive strength increased by 46.2% compared with the 3 d, and the 28-d compressive strength increased by 34.2% compared with the 7 d. Group MRAC-0.36 showed the smallest increase in compressive strength. The 7-d compressive strength is 25.3% higher than that at 3 d, and the 28-d compressive strength is only 23.1% higher than that at 7 d. The compressive strength of group MRAC-0.30 also increased significantly, the 7 d compressive strength increased by 35.5% compared to the 3-d compressive strength, and the 28 d compressive strength increased by 41.9% compared to the 7-d compressive strength. The early strength of MRAC develops rapidly, and the compressive strength at 3 d can reach approximately 50%. With the progress of cement hydration, the later strength value could still stably increase. Additionally, the compressive strength values at 28 d of the four groups range from 22.2 MPa to 37.9 MPa, which can meet the requirements of general engineering. The results also showed that the dispersion of the strength values of each group was generally small, indicating that the components were becoming an entirety by cement colloid, which could maintain good homogeneity.

**Figure 5:**
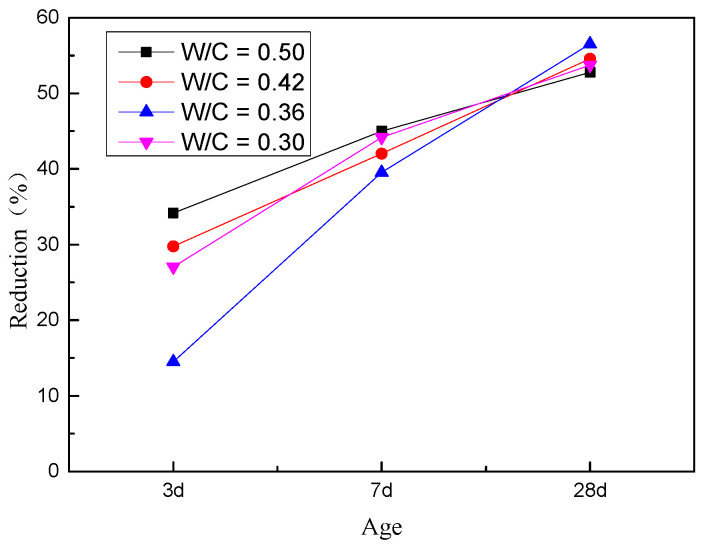
Reduction of compressive strength at different ages.

Combining Figure 4 and Figure 5, it can be found that the difference between the compressive strength of MRAC and the reference value gradually increases with increasing age. This showed that the deterioration of the properties of MRA has a great impact on the compressive strength of concrete. It is not appropriate to design RAC, especially MRAC, according to ordinary concrete design methods. The formula of the W/C calculation of natural aggregate must be revised before it could be used for MRA; otherwise, it would cause a significant drop in compressive strength and quality problems of engineering.

Figure 5 also revealed that comrapred with reference group, the 3-d compressive strength of the four groups of MRAC decreased in the range of 14–34%, and the decrease at 7 d was 40–45%. By 28 d, the decrease was more than 50%, and the distribution of decline in the early stage was more scattered, while it was more concentrated in the later stage. It showed that the strength of the weak particles in the MRA is the key factor that determines the strength of the concrete. The difference in the aggregate has a greater impact on the strength than the W/C. This also indirectly reflects that although the method of reducing the W/C can increase the compressive strength to a certain extent, it still cannot narrow the gap of properties between MRA and natural aggregate. In the late stage of hydration, the MRA and cement mortar are becoming an entirety with good homogeneity. The load on the weak aggregate was shared by the cement slurry, and the influence of the difference in the aggregate was reduced. Therefore, the decrease in the compressive strength of MRAC is consistent.

**Figure 6:**
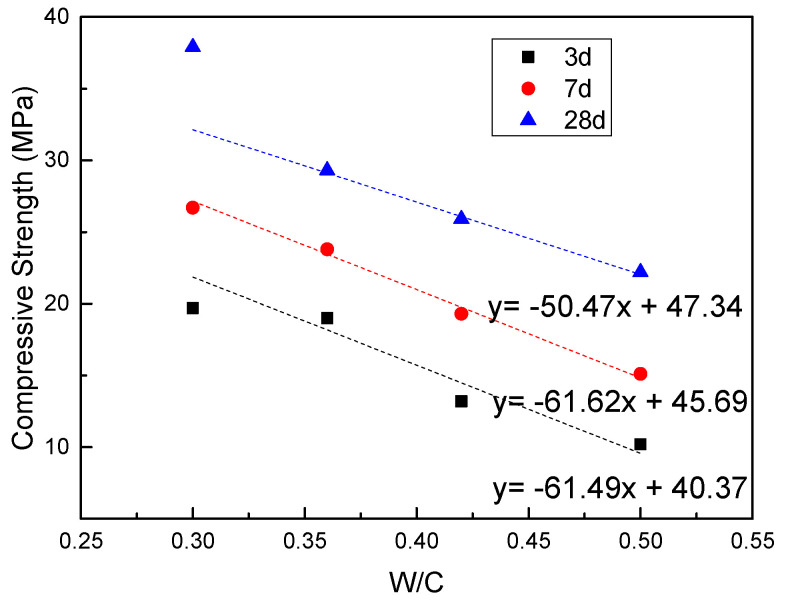
The Effect of W/C on Compressive Strength.

Figure 6 showed the effect of W/C on the compressive strength of MRAC. For ordinary concrete, the W/C and compressive strength show a certain linear relationship. Similarly, in the high W/C area, the compressive strength of MRAC also decreases with increasing W/C and presents an obvious linear relationship. When the W/C is 0.3, the compressive strength value deviates from the fitted curve. At the ages of 3 d and 7 d, the compressive strength of MRAC-0.30 is below the curve. This is because in the early stage of hydration, the strength of concrete is mainly affected by MRA. Weak aggregates, such as broken bricks and tiles, were not covered by enough Calcium Silicate Hydrated gel (CSH gel) in the mortar, which had a certain impact on the strength of the concrete. At 28 d, the compressive strength of MRAC-0.30 is above the curve. It showed that with increased cement hydration, the strength of the mortar continued to increase. When strengthening the weak aggregate, the mortar could also bear part of the load, and then the strength would be higher than the predicted value.

### 3.2. Results of Splitting Tensile Strength Test

**Figure 7:**
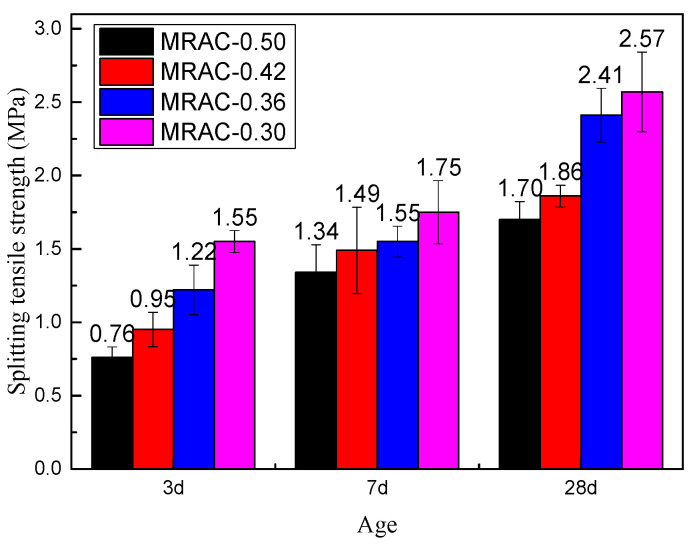
Splitting Tensile Strength of MRAC at Different Ages.

As shown in Figure 7, similar to the results of the compressive strength, with increasing age, the splitting tensile strength of each group of MRACs continuously improved. The test results showed that for the test group with a higher W/C, the splitting tensile strength growth rate was higher at an early age. The 7 d splitting strength of group MRAC-0.50 was 76.3% higher than that of the 3 d splitting strength, and the increase ratio of group MRAC-0.42 was 56.8%. However, in the later stage, the strength increase ratio was small. The 28 d splitting strength of group MRAC-0.50 was only 26.9% higher than that at 7 d, and the increase ratio of group MRAC-0.42 was only 24.8%. However, the trend of the low W/C test group was opposite, and the increase ratio of splitting tensile strength was lower at an early age. The 7 d splitting strength of group MRAC-0.36 was 27.0% higher than that of the 3-d splitting strength, and the increase ratio of group MRAC-0.30 was 12.9%. In the later stage, the strength growth ratio was higher. The 28 d splitting strength of group MRAC-0.36 was 55.5% higher than that at 7 d, and the increase ratio of group MRAC-0.30 was 46.9%. Moreover, compared with the results of the compressive strength test, the splitting strength values are more discrete, indicating that the splitting tensile strength of MRAC was more dependent on the basic properties of the MRA. The bonding ability of old mortar and cementing materials, the bonding ability of aggregate and cementing material, and the strength of discarded bricks would greatly affect the splitting tensile properties of MRAC.

### 3.3. Results of Elastic Modulus Testing

**Figure 8:**
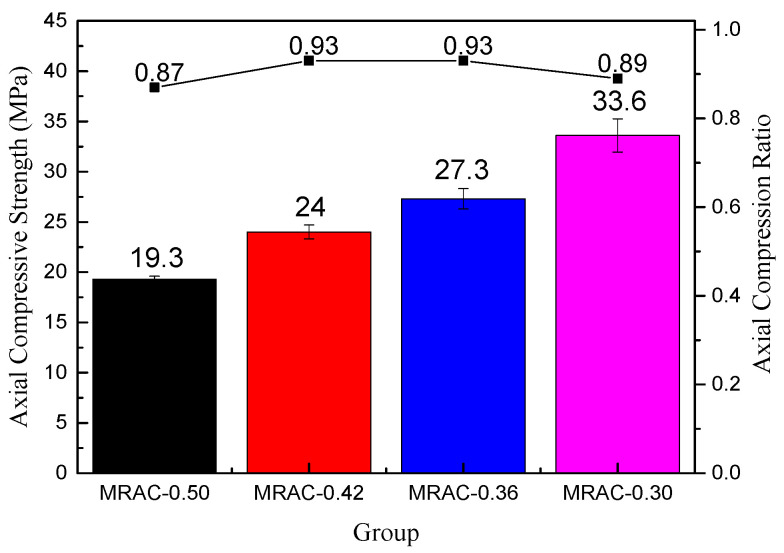
Axial Compressive Strength and Axial Compression Ratio of MRAC.

Figure 8 showed that with decreasing W/C, the axial compression strength of the MRAC increases. This is consistent with the law of the compressive strength test and split strength test. It can also be seen from Figure 8 that the axial compression ratios of the four groups of MRAC were all above 0.87. Compared with the Chinese standard of the Code for Design of Concrete Structures (GB 50010-2010), which requires the minimum axial compression ratio of ordinary concrete below C40 to be 0.67, the test results are relatively safe, indicating that it is feasible to apply MRAC to actual projects.

**Figure 9:**
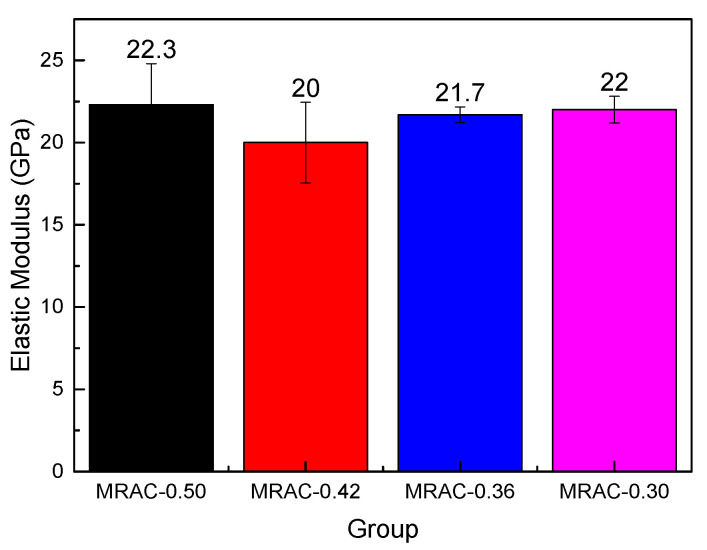
Elastic Modulus of MRAC under different W/C.

The results in Figure 9 indicated that with increasing W/C, the elastic modulus of the MRAC has a gradually decreasing trend, and the degree of dispersion increases accordingly. This may be because under the low W/C, the amount of cement was relatively large, and more gels would be formed after hydration, which can better cement the MRA into an entirety. However, when the W/C was relatively high, the amount of cement was low, and the excess water easily left pores in the concrete after evaporation or absorption. Additionally, as a brittle material, discarded brick aggregate would easily form a weak area if there was not enough dense cement mortar, which will affect the mechanical properties of MRAC. Therefore, in this experiment, the dispersion degree of the high W/C test group was significantly greater than that of the low W/C test group.

### 3.4. Results of MIP Test

**Figure 10:**
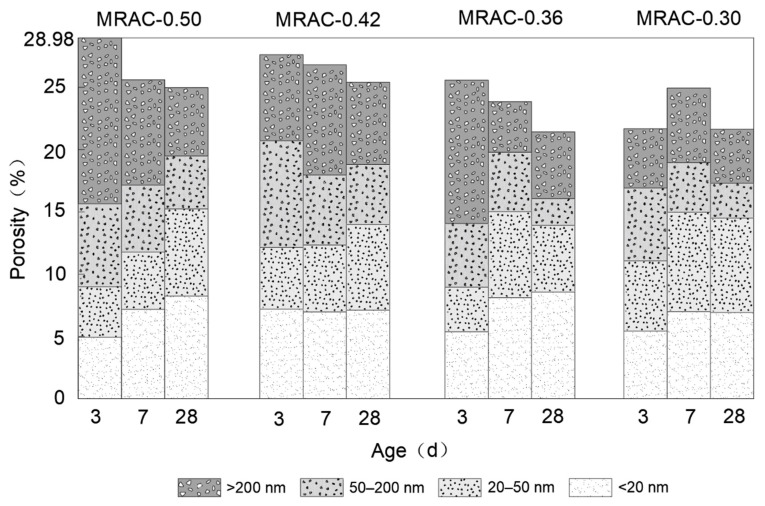
Internal Pore Size Distribution of MRAC at Different Ages.

Figure 10 showed the percentage of pore size distribution in MRAC under different W/C ratios at three ages. The pore structure of different pore diameters has different effects on the durability of concrete. According to the degree of influence, it can be divided into four types, namely, harmless pores (<20 nm), less harmful pores (20–50 nm), harmful pores (50–200 nm) and the most harmful holes (>200 nm). Figure 10 showed that the most harmful pores and harmful pores have a negative impact on the durability of concrete, accounting for 49–69% at 3 d, which is more than the sum of less harmful pores and harmless pores. At the age of 7 d, the total proportion of most harmful pores and harmful pores was between 37% and 44%. Due to the filling of hydration products, some of the most harmful pores and harmful pores have begun to change to less harmful and harmless pores. Taking the group MRAC-0.36 as an example, the proportion of less harmful holes increased by 15%, and the proportion of harmless holes increased by 14%. At the age of 28 d, the degree of cement hydration was relatively high, and the total proportion of less harmful pores and harmless pores could basically reach approximately 60%.

In summary, as age increased and cement hydration continued, the large pores in each group of MRAC were gradually filled with hydration products. The total porosity showed a trend of declining continuously. At the age of 28 days, the porosity of the four groups of specimens was between 21% and 26%, which was higher than that of ordinary concrete. The proportion of most harmful pores and harmful pores cannot be further reduced, which was mainly because discarded brick is a kind of porous material that contains many closed pores. For cementitious material, it was difficult to enter these closed pores. There are also many cracks and pores between the old mortar and the MRA. It is difficult for hydration products to reach this part of the area through the old mortar. Therefore, compared with ordinary concrete, there are still a certain proportion of most harmful pores and harmful pores inside the MRAC at the age of 28 d. Pores such as these would have a certain impact on the durability of concrete, which should be considered in actual engineering.

### 3.5. Results of XRD Test

**Figure 11:**
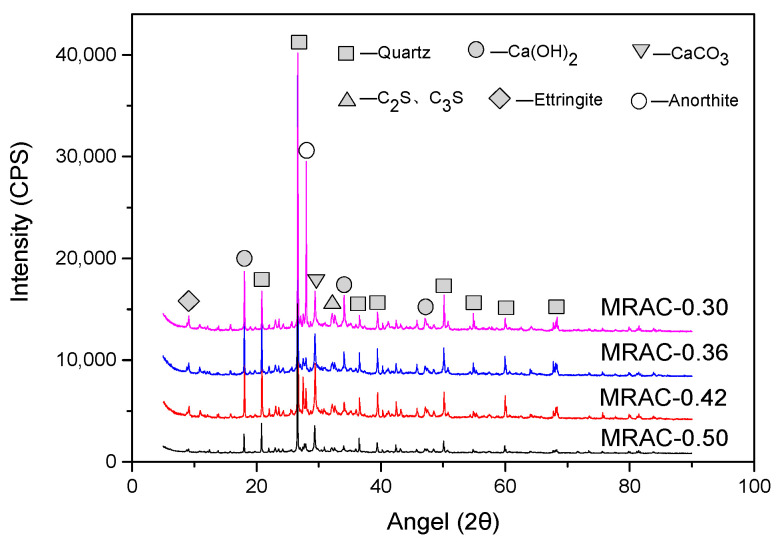
X-ray Diffraction Pattern of MRAC at 28 days.

Figure 11 indicated that the main mineral components of the MRAC include SiO_2_, Ca(OH)_2_, CaCO_3_, C_2_S, C_3_S, ettringite, and anorthite. Among these, the diffraction peak of SiO_2_ was the highest, indicating that as the main mineral component of sand, it was also the most abundant in MRAC. The diffraction peaks of anorthite were much higher in test groups MRAC-0.42 and MRAC-0.30, while it is average in groups MRAC-0.50 and MRAC-0.36, indicating that anorthite may be an impurity component contained in MRA. C_2_S and C_3_S are unhydrated cement clinkers, and their peak intensity gradually decreases from top to bottom in the four spectral lines. In the group MRAC-0.50, the diffraction intensity of C_2_S and C_3_S was 292, while in the group MRAC-0.30 the diffraction intensity was 384, indicating that C_2_S and C_3_S still had a certain amount of residual after 28 days. The lower the W/C, the more C_2_S and C_3_S were residual. It also indicates that the hydration reaction of the cement would continue after the standard curing age, and the properties of MRAC could be improved to a certain extent.

Ca(OH)_2_ and ettringite were the products of cement hydration. The crystal state and growth direction of these hydrated products would have a significant impact on the macroscopic properties of concrete. In Figure 11, the diffraction peak of ettringite decreases with increasing W/C, indicating that its content is basically positively correlated with the cement content. For Ca(OH)_2_, except for the lower peak value in the group MRAC-0.50, there was no obvious pattern in the groups MRAC-0.42, MRAC-0.36, and MRAC-0.30. This may be because there were still some C_2_S and C_3_S inside MRAC that did not convert into Ca(OH)_2_ and CSH gel. Figure 11 also shown that the diffraction intensities of CaCO_3_ of the four groups were different. The peak value of group MRAC-0.42 was the highest, while that of the group MRAC-0.50 was the lowest. The peak values of the two groups MRAC-0.36 and MRAC-0.30 were similar. Considering that the samples were tested immediately after curing, the impact of carbonization was negligible. Therefore, it is believed that calcium carbonate, like anorthite, may be an impurity component in MRA.

### 3.6. Results of SEM Test

**Figure 12:**
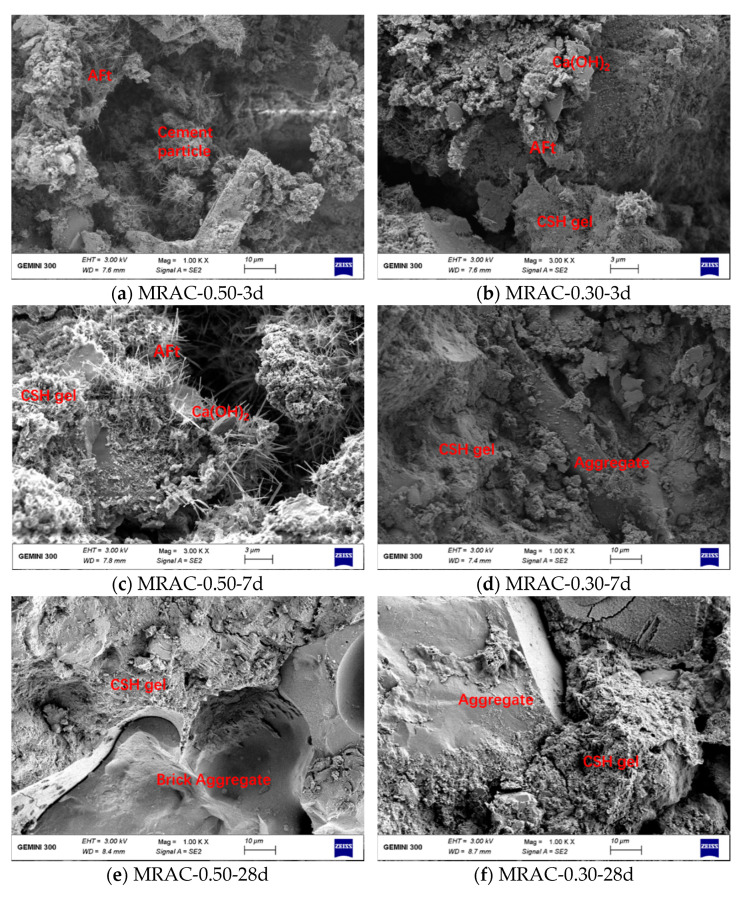
SEM Pictures of MRAC. (Note: AFt represents high-sulfur hydrated calcium sulfoaluminate).

Figure 12 compared the microstructure of the two experimental groups, MRAC-0.50 and MRAC-0.30 at different ages. It can be seen from Figure 12a that at the age of 3 d, the cement particles in group MRAC-0.50 were distributed in pores and cracks as clusters. Some cement particles just started to hydrate. Needle-like ettringite appeared on the surface with a small amount of CSH gel, while no Ca(OH)_2_ was formed. Figure 12b showed that the degree of hydration in group MRAC-0.30 was higher than that in MRAC-0.50. A certain amount of CSH gel and flaky Ca(OH)_2_ crystals were formed, and the hydration products at both ends of the cracks began to be close to each other. Generally, in the early stage of age, due to the small amount of hydration products, it was not enough to effectively fill the pores and wrap the aggregate. Therefore, the strength of MRAC was low, and the pore size of the internal pore structure was also larger. At the age of 7 d, a certain amount of CSH gel and a small amount of Ca(OH)_2_ crystals began to appear in group MRAC-0.50, while in group MRAC-0.30, a large piece of gel had formed, which completely wrapped the aggregate. The hydration rate of the low W/C group was faster than that of the high W/C group. At 28 d age, the degree of cement hydration increases. The aggregates of the groups MRAC-0.50 and MRAC-0.30 were surrounded by CSH gel and in close contact with each other without gaps. As a typical weak aggregate, the discarded brick shown in Figure 12e was easily damaged and had adverse effects on concrete. After the CSH gel was bonded and filled, the porous aggregate and the surrounding cement mortar became an entirety. The mortar will bear part of the load when underloaded, and then the adverse effects from weak aggregate are reduced.

**Figure 13:**
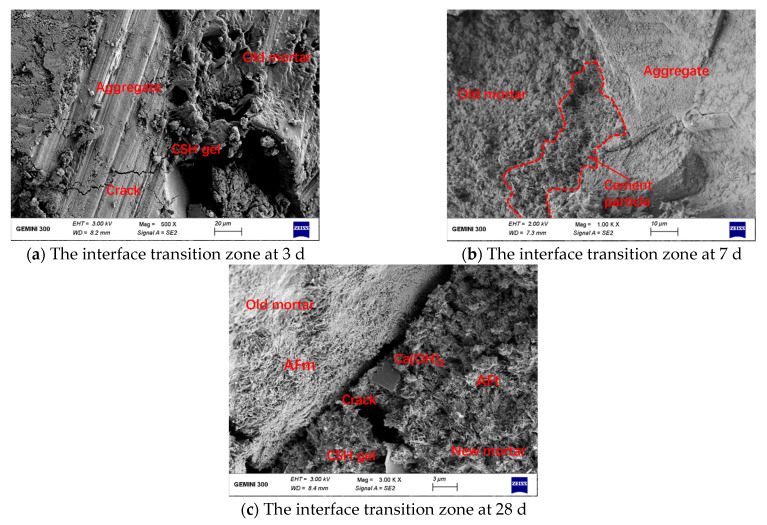
The Interface Transition Zone of MRAC.

The interface transition zone has always been regarded as the weakest zone in concrete due to its loose structure and easy cracking. It is an important factor that could affect the mechanics and durability of concrete. Therefore, it is necessary to study the properties of the interface transition zone inside MRAC. Figure 13 showed the development of the interface transition zone with age. It can be seen from Figure 13a that many microcracks had appeared on the surface of the MRA on the left, and the maximum width was close to 2 μm, indicating that part of the strength of the aggregate had been lost. The old mortar on the right belonged to the type of loose porous structure, which was unbonded with the MRA. Moreover, the gel shrank, and the cement lost its chemical activity, indicating that the strength of the cement mortar was reduced and that the cement mortar could be peeled off easily from the aggregate. This means that it would be difficult for the cement mortar to resist the invasion of harmful substances. Figure 13b showed that the cement particles attached to the MRA began to hydrate at the age of 7 days. In this picture, the old mortar was relatively dense and had fewer holes, but there were still approximately 10 μm gaps between the MRAs. As the cement particles fell into the gap and began to hydrate, the voids were filled, and the crack width decreased continuously. Figure 13c showed that the interface transition zone of MRAC at 28 d of age. The left side of the picture was the old mortar. The surface of the old mortar was covered with foil-like monosulfide hydrated calcium sulfoaluminate (AFm). There was no needle-like ettringite in the picture, indicating that AFt had been converted to AFm. Thus, the old mortar had no hydration activity. There was the hydration product growing toward the old mortar on the right side. The gap between the old mortar and new mortar was less than 1 μm. The hydrated products contained flaky Ca(OH)_2_, needle-like ettringite, and flocculent CSH gel with a disordered distribution. There were still some microholes and microcracks in the interface transition zone. As the age changed, we found that the property improvement in the interface transition zone of MRAC was mainly related to the hydration process of the cement slurry. Through the growth and filling of hydration products, the original cracks and pores continuously decreased, and the density of the interface transition zone increased. The bonding strength of the gel between the old mortar and the MRA and the old mortar and new mortar was improved. Thus, the properties of MRAC were improved as well.

## 4. Conclusions

In this paper, using MRA as the raw material, four groups of MRAC under different W/C ratios were designed and prepared to study the mechanical properties and microstructure. The experimental results and discussion lead to the following conclusions:

1. Using MRA to prepare MRAC, the maximum 28 d compressive strength can reach 37.9 MPa, which can meet the requirements of general engineering.
2. Compared with ordinary concrete, the compressive strength of MRAC with the same W/C is greatly reduced, and the drop is over 50% at 28 d. In contrast, the axial compression ratio of MRAC is above 0.87, which means that the safety is better. Therefore, special attention should be paid to the influence of MRA when designing the mix ratio.
3. The compressive strength of MRAC linearly decreases with increasing W/C when the W/C is higher than 0.30. When the W/C is lower than 0.30, the compressive strength of MRAC at the early stage is lower than the predicted value but is higher at the later stage. The reason is that the weakness of MRA affects the early strength of MRAC. The properties of MRA could be strengthened by cement slurry over time.
4. The XRD results showed that the hydration rate of cement in MRAC is slow. However, the additional water absorbed by the MRA can act on the unhydrated cement clinker to form an internal curing mechanism, which is beneficial to the long-term development of the properties of the MRA.
5. The increase in cement content can improve the mechanical properties of MRA. The main reasons are as follows: (i) Filling harmful pores effectively and then reducing the pore size of the pore structure; (ii) The wrapping of the formed hydration products strengthens the MRA more effectively.

## Figures and Tables

**Table 1:** Technical Indicators of MRAs.

Technical Index	MRA-1	MRA-2	MRA-3
Brick Content (%)	12.5	21.9	-
Crush Index (%)	18	21	22
Apparent Density (kg/m^3^)	2500	2480	2410
Void Ratio (%)	52	53	49
2 h Water Absorption (%)	7.9	8.0	10.1
24 h Water Absorption (%)	8.7	9.1	13.2
Chloride Ion Content (%)	0.014	0.017	<0.005
Sulfate Content (%)	0.012	0.049	0.023

Note: The particles <4.75 mm in MRA-3 are too fine to determine the brick content.

**Table 2:** Basic Properties of Standard Cement.

Items	Test Results
Fineness (≤0.075 μm) (%)	1.0
Specific Surface Area (m^2^/kg)	340
Standard Consistency (%)	25.4
Density (kg/m^3^)	3150
Stability (Reye’s Method) (mm)	0.3
Initial Setting Time (min)	175
Final Setting Time (min)	225
Flexural Strength (MPa), 3 d	6.2
Flexural Strength (MPa), 28 d	9.4
Compressive Strength (MPa), 3 d	27.6
Compressive Strength (MPa), 28 d	52.8

**Table 3:** Mix Ratio of MRAC (kg/m^3^).

Group	Total Water	Cement	MRA-1	MRA-2	MRA-3	Water Reducing Agent
MRAC-0.50	327.3	336.2	635.3	95.3	1022.9	6.1
MRAC-0.42	323.0	397.2	618.2	92.7	995.2	7.1
MRAC-0.36	317.9	469.0	598.0	89.7	962.7	8.4
MRAC-0.30	311.9	553.4	574.2	86.1	924.4	10.0

## Data Availability

Data is contained within the article.
